# Genetic transformation of *Primula sieboldii* using *Agrobacterium rhizogenes* and whole-plant regeneration from transgenic hairy roots

**DOI:** 10.3389/fpls.2025.1623387

**Published:** 2025-07-25

**Authors:** Karol Gad, Cuong Nguyen Huu, Sylvia Plaschil, Christian Kappel, Michael Lenhard

**Affiliations:** ^1^ Institute of Biochemistry and Biology, University of Potsdam, Potsdam-Golm, Germany; ^2^ Institute for Breeding Research on Horticultural Crops, Julius Kühn Institute - Federal Research Centre for Cultivated Plants, Quedlinburg, Germany

**Keywords:** primrose, genetic engineering, plant biotechnology, plant tissue culture, Agrobacterium-mediated plant transformation

## Abstract

*Primula sieboldii* E. Morren is a widely cultivated ornamental plant with horticultural and pharmacological value. However, the lack of a developed transformation system has limited genetic studies and biotechnological applications of this species. In this study, we established a transformation method using *Agrobacterium rhizogenes* for the genetic manipulation of *Primula sieboldii*. The protocol consists of two stages: initial hairy root transformation and subsequent whole-plant regeneration from transgenic hairy roots through somatic embryogenesis. Comprehensive molecular analyses confirmed the stable integration and expression of various transgenes from the root-inducing (Ri) plasmid and the binary vector carrying the *RUBY* reporter in independent transgenic lines, as well as the stable germline transmission of the transgene to progeny. The protocol is effective, with 5% of treated explants successfully forming transformed hairy roots expressing the *RUBY* reporter, from which transgenic plants were regenerated. The established method provides a valuable tool for genetic and molecular studies of heterostyly and the self-incompatibility system in the genus *Primula*, while also offering practical applications in molecular breeding and plant biotechnology. Additionally, hairy root cultures provide a platform for metabolic engineering and the exploration of biologically active secondary metabolites with pharmacological applications.

## Introduction

The genus *Primula* L., commonly known as primroses, is represented by approximately 500 species, most of which naturally occur in the Northern Hemisphere (Richards, 2003). Due to their early spring bloom and colorful, attractive flowers, many *Primula* species have gained popularity in cultivation, making them important ornamental plants in horticulture.

*P. sieboldii* is a perennial species native to Northeast Asia, that has been cultivated for centuries, with its breeding process involving crossing and selection has resulted in over 300 cultivars with diverse flower colors and shapes (Yoshioka et al., 2005; Honjo et al., 2008). Additionally, this species has been used for interspecific and intersection crosses, which resulted in hybrid plants regenerated *in vitro* using embryo rescue techniques (Kato et al., 2001; Kato and Mii, 2000).

Hairy root cultures are commonly used in plant biotechnology for producing valuable secondary metabolites, recombinant proteins, and molecular breeding (Ono and Tian, 2011). Transformed hairy roots are induced upon inoculation with *A. rhizogenes* – a soil bacterium that contains the root-inducing (Ri) plasmid with a fragment of transfer DNA (T-DNA), which can be stably integrated into the host plant genome. Moreover, *A. rhizogenes* harboring the Ri plasmid can be used along with binary vectors for expressing foreign genes in hairy roots (Bahramnejad et al., 2019). This type of culture is suitable for producing large quantities of secondary metabolites, which can often be scaled up to allow growth in bioreactors (Gutierrez-Valdes et al., 2020; Morey and Peebles, 2022). Furthermore, transformed hairy roots can be induced to form shoots and regenerate whole plants (Jedličková et al., 2024).

There is a growing interest in the characterization and potential use of secondary metabolites from plants within the genus *Primula*. Some species have been used as medicinal herbs for centuries, and modern studies have demonstrated their diverse pharmacological activities. For example, *P. vulgaris* Huds. flower extract has been shown to have anticancer properties (Demir et al., 2018). Additionally, leaf extracts from *P. elatior* (L.) Hill. and *P. veris* L. exhibited good results in terms of affinity for the GABA(A)-benzodiazepine receptor (Jäger et al., 2006), and anti-influenza virus activity (Eliopoulos et al., 2022). Flavones from *Primula veris* subsp. *macrocalyx* appear to have pharmacological effects such as antioxidant, anti-inflammatory effects and antifungal properties (Li et al., 2019).

Among the various secondary metabolites, saponins, particularly sakurasosaponin, which is found in the roots of *P. sieboldii* are of significant therapeutic interest (Ikenishi et al., 1976). Notably, this compound extracted from the roots of this species exhibits promising medicinal properties, including anticancer activity. Sakurasosaponin activates the AMPK pathway, inducing cell autophagy and inhibiting the cell proliferation of non-small cell lung cancer (NSCLC) (Seo et al., 2023). Additionally, sakurasosaponin inhibits the expression of the androgen receptor, which induces cell death in prostate cancer cells (Song et al., 2020). These findings highlight the potential of *P. sieboldii* as a medicinal plant, offering valuable biologically active secondary metabolites for pharmacological applications, including new drug development.

Like many other species of primroses, *P. sieboldii* has heterostylous flowers with two morphs that differ in the reciprocal positions of stigma and stamens, promoting outcrossing (Kappel et al., 2017). The S-morph (thrum) is characterized by a short style and high positions of anthers, while the L-morph (pin) has a long style and a low position of anthers. These floral characteristics are controlled by the *S*-locus supergene, a hemizygous region of the S-morph. Several predicted genes, such as *CCT^T^*, *CYP734A50*, *GLO2*, *KFB^T^*, and *PUM^T^* have been identified (Li et al., 2016). Heterostyly in primroses was mentioned by Charles Darwin (1877), and the genus *Primula* is a prime model for studying this complex floral adaptation (Nowak et al., 2015; Potente et al., 2022).

Some of the *Primula S*-locus genes have only been partially characterized. It has been shown that *CYP734A50* controls style length and female self-incompatibility (Huu et al., 2022; 2016), while *GLO2* affects anther position (Huu et al., 2020). Previous studies have relied on identifying naturally occurring mutants from wild or cultivated populations, or inducing mutagenesis followed by plant screening. These methods suffer from several disadvantages, including randomness, off-target mutations, and challenges in identifying the desired mutations. Another approach for non-model plants is virus-induced gene silencing (VIGS), but this system is transient, resulting in only temporary gene silencing with variable efficiency (Wege et al., 2007). Against this background, a method for stable transformation would be highly desirable, because it would allow investigating the role of the remaining genes and enable functional studies of the encoded proteins, e.g. using epitope-tagged versions.

This study presents the first protocol for establishing an *A. rhizogenes*-mediated genetic transformation system for *P. sieboldii*. The described method provides a valuable tool for enabling further research on this species, facilitating genetic studies and applications in biotechnology.

## Materials and methods

### Plant culture media

The plant culture media were prepared as shown in **Table 1**. All media were sterilized by autoclaving at 121 °C under pressure of 2 atm for 15 minutes. Thermolabile substances were sterilized by filtration through a 0.2 µm membrane filter (Whatman Schleicher & Schuell) and added after autoclaving.

**Table 1:** Composition of plant culture media.

Medium	Composition	Purpose
MS10	½ Murashige & Skoog (1962) basal medium including Gamborg B5 vitamins (Gamborg et al., 1968) (Duchefa, the Netherlands), 1% (w/v) sucrose, 0.1 mg/L GA_3_, 8g/L agar (Sigma−Aldrich, A7921, CAS 9002−18−0), pH 5.8	For seed germination
MS20	Murashige & Skoog basal medium including Gamborg B5 vitamins, 2% (w/v) sucrose, 8g/L agar, pH 5.8	General-purpose medium for *in vitro* culture; supports hairy root growth, somatic embryo development, and root formation
Co-cultivation medium	Murashige & Skoog basal medium including Gamborg B5 vitamins, 3% (w/v) sucrose, 5 g/L PVP average mol wt 40,000 (Sigma-Aldrich, USA), 200 μM acetosyringone, 3 g/L gelrite (Duchefa, the Netherlands), pH 5.8	For *A. rhizogenes*-mediated transformation
Callus-inducing medium	Murashige & Skoog basal medium including Gamborg B5 vitamins, 3% (w/v) sucrose, 2 mg/L NAA, 0.2 mg/L TDZ, 5 g/L PVP, 3 g/L gelrite, pH 5.8	For induction of callus and somatic embryo development

### Establishment of plant tissue culture

*P. sieboldii* seeds used in this study were purchased from the seed breeder and supplier Jelitto Staudensamen GmbH (Germany). Subsequently, plants were propagated from these seeds and used for crosses. All seeds derived from a single cross were used for all experiments to ensure genetic consistency. Wild-type control plants (non-transformed), derived from the same seed batch, were germinated *in vitro* and transferred to the greenhouse alongside the transformants.

The culture establishment and transformation were carried out under sterile conditions in a laminar flow hood. Seeds were sterilized by washing in 70% ethanol, followed by shaking in a bleach solution (5% active chlorine) containing 0.1% Triton X-100 for 3 minutes. The seeds were then rinsed three times with 100% ethanol. After removing the ethanol, the seeds were air-dried and placed on MS10 medium supplemented with 0.1 mg/L gibberellic acid (GA_3_). Seed germination was typically observed within 7 days, and then seedlings were transferred to MS20 medium. Plants were grown under long-day conditions, with 16 hours of light and 8 hours of darkness at 20°C, with a light intensity of 50–60 µmol m^-2^ s^-1^. The compositions of all media used in this study are listed in **Table 1**.

### Bacteria preparation

*A. rhizogenes* A4 was obtained from the Spanish Type Culture Collection (CECT) at the University of Valencia and cultured at 26°C on TY (5 g/L tryptone, 3 g/L yeast extract, 0.9 g/L CaCl_2_ x 2H_2_O, pH 7.2, and optionally 15 g/L agar) following the growth conditions recommended by the CECT. The wild-type *A. rhizogenes* strain A4 carries the native RiA4 plasmid, which is essential for hairy root induction, was maintained under aseptic conditions without antibiotic selection.

The preparation of electrocompetent *A. rhizogenes* A4 was performed following a modified version of a previously published protocol (Wise et al., 2006). A freeze-dried culture of *A. rhizogenes* A4 was resuspended in 0.3 mL of liquid TY medium, streaked on TY agar medium, and incubated at 26°C for 2 days. A single colony was inoculated into 50 mL of medium and incubated with shaking (220 rpm) at 26°C for 2 days. The optical density (OD_600_) was measured on an Eppendorf BioPhotometer. To initiate the main culture, 17 mL of the saturated culture (OD_600_: 1.8-2.0) was transferred to 250 mL of fresh TY medium, resulting in an initial OD_600_ of 0.1. The culture was incubated at 26°C with shaking at 220 rpm, and the OD_600_ reached 0.4 after approximately 5 hours. The cells were then cooled on ice for 15 minutes and centrifuged at 4,000 rpm for 10 minutes at 6°C. The supernatant was discarded, and the cell pellet was resuspended in 30 mL of cold sterile water. The suspension was centrifuged again under the same conditions, and the supernatant was discarded. The cells were then resuspended in 10 mL of 10% glycerol and centrifuged at 4,000 rpm for 10 minutes at 6°C. After discarding the supernatant, the cells were resuspended in 1 mL of 10% glycerol. Aliquots of 20 μL were prepared and frozen in liquid nitrogen, then stored at -80°C until use.

The 35S:RUBY plasmid was purchased from Addgene (plasmid #160908) and was originally a gift from Yunde Zhao. The strain *A. rhizogenes* A4 was transformed with 35S:RUBY using a standard electroporation protocol for *Agrobacterium* with the MicroPulser Electroporator (Bio-Rad) at 2.5 kV, 400 Ω. After transformation, bacteria were selected on TY medium supplemented with spectinomycin (50 mg/L). Prior to the plant transformation experiment, bacteria were streaked on TY agar medium containing spectinomycin (50 mg/L) and cultured for 2 days. A single colony was then inoculated into 5 mL liquid TY medium and grown for 2 days. A 100 µL of this culture was inoculated into fresh medium 100 mL one day before transformation. On the day of transformation, the culture reached an OD_600_ of 0.8, was centrifuged at 6,000 rpm for 15 minutes, and the cells were resuspended in liquid MS20 medium supplemented with 200 μM acetosyringone to an OD_600_ of 0.1.

### Hairy root transformation

The transformed *A. rhizogenes* A4 strain carries both its native RiA4 plasmid and the binary vector containing the 35S:RUBY construct, enabling simultaneous co-transformation of both T-DNAs. The binary vector 35S:RUBY harbors the visual reporter *RUBY*. The 35S:RUBY T-DNA contains *CYP76AD* (*P450 oxygenase*), *DODA (L-DOPA 4,5-dioxygenase*), and *GT* (*glucosyltransferase*) genes, under the control of the Cauliflower Mosaic Virus (CaMV) *35S* promoter (**Supplementary Figure S1**). These genes encode enzymes that convert tyrosine into vividly red betalain, allowing the detection of transformation events through the production of red pigment in the transformed cells (He et al., 2020). Explants for transformation were taken from mature plants grown in tissue culture (approximately 3 months old). Lateral roots were cut into small segments approximately 0.5 to 1 cm and pre-cultured on callus-inducing medium in the dark at 20°C for 20 days to induce embryogenic callus. The explants were then immersed in a bacterial suspension (OD_600_: 0.1) and incubated with gentle shaking (25 rpm) at room temperature for 10 minutes. Afterward, the explants were blot-dried and placed on co-cultivation medium and kept in the dark at 20°C for 2 days. To remove excess *A. rhizogenes*, the explants were washed three times for 10 minutes each in distilled sterile water supplemented with 350 mg/L timentin (ticarcillin disodium/clavulanate potassium) and 0.1% PPM (Plant Preservative Mixture, Plant Cell Technology, Inc., USA). The blot-dried explants were then placed on M20 medium supplemented with 250 mg/L timentin and 0.1% PPM. Hairy roots were induced on hormone-free MS20 medium, which was refreshed every 3 weeks until root development was observed. Typically, transgenic hairy roots with thick morphology started to emerge directly from infection sites after 4–6 weeks. Those expressing the *RUBY* reporter were easily distinguishable due to the visible pigmentation resulting from the activity of enzymes involved in the betalain biosynthetic pathway.

### Whole-plant regeneration

Transformed hairy roots producing red betalain were cut from the primary explants and were considered independent transformation events if they originated from different explants. Small root segments approximately 0.5 to 1 cm in length, were placed on callus-inducing medium and cultured in the dark for 4–6 weeks to promote embryogenic callus formation. Continued culture on the same medium enhanced somatic embryo formation from the previously induced callus tissue. The explants with well-developed somatic embryos were then transferred to MS20 medium (without plant growth regulators) and exposed to light conditions to facilitate embryo maturation. Subsequently, germinated embryos were separated and cultured on MS20 for an additional 2–4 weeks. Finally, the plantlets with developed roots were transferred to sterile jars containing MS20 and cultured for approximately 9 weeks before being moved to the greenhouse. The list of all media composition is provided in **Table 1**.

### Plant hardening

Plants derived from tissue culture with well-developed roots were acclimated to greenhouse conditions. Their roots were thoroughly washed to remove any agar residue before careful transplantation into a substrate composed of peat, clay, and perlite (pH 5.8). Initially, the plants were covered with a plastic dome for one week to increase humidity and facilitate their adaptation to the greenhouse environment. They were grown under long-day conditions with 16 hours of light and 8 hours of darkness at 20°C. Lighting was provided by CHD Agro 400 ceramic metal halide lamps installed in closed fixtures (DH Licht GmbH, Germany), delivering a photosynthetic photon flux density (PPFD) of approximately 416 µmol m^-2^ s^-1^ at canopy level.

### Genetic crosses

Before pollination, the flowers were emasculated and hand-pollinated by applying pollen from mature anthers directly onto the stigma under a binocular microscope. Mature capsules were harvested before dehiscence 6 weeks after pollination. The seeds were cleaned and stored at 4°C before sowing.

### DNA isolation

Genomic DNA was isolated from young leaves using the CTAB (cetyltrimethylammonium bromide) method. DNA quality was assessed using the DeNovix DS-11 spectrophotometer, and its concentration was measured using the Qubit™ dsDNA Assay Kit (Invitrogen) according to the manufacturer’s instructions.

### PCR

DNA used for PCR was isolated from leaf tissue of non-transformed and transformed plants maintained in tissue culture. Controls included wild-type *P. sieboldii*), 35S:RUBY plasmid, *A. rhizogenes* A4 carrying the 35S:RUBY plasmid, and no template control to confirm the absence of contamination and non-specific amplification. PCR was performed using MyTaq™ DNA Polymerase (Meridian Bioscience) according to the manufacturer’s instructions. Amplified PCR products were analyzed by electrophoresis on an agarose gel containing ethidium bromide and visualized under UV light. Oligonucleotide sequences are listed in **Supplementary File S1**.

### Whole-genome sequencing and identification of T-DNA insertions

DNA was extracted from leaf tissue of three independent transgenic lines and one wild-type plant all grown in the greenhouse following post-hardening stage, was sent for Illumina whole-genome sequencing to Novogene Europe (Munich). Sequencing reads were mapped against the two construct sequences using BWA-MEM (Li, 2013), and further processed with SAMtools (Li et al., 2009). Transition sequences between the constructs and the *P. sieboldii* genome, which were absent in the wild-type sample, were reconstructed based on unmapped read pairs and partially mapped reads. High-throughput sequencing raw data have been deposited in the NCBI SRA under project number PRJNA1191157.

To estimate the number of T-DNA insertion sites, we used genome coverage as a reference. Conserved single-copy genes in the *P. veris* genome (GCA_963682055.1_ddPriVeri1.1, NCBI) were identified using BUSCO v5.8.2_cv1 with the embryophyta_odb12 lineage dataset. Coding sequences (CDS) of all identified single-copy genes were extracted individually. Illumina whole-genome sequencing reads from *P. sieboldii* were aligned to these CDS using BWA-MEM. Coverage for each CDS was calculated with samtools coverage, and the meandepth values were extracted. To avoid bias from absent or highly divergent genes, only CDS with meandepth > 0 (i.e., with at least some aligned reads) were retained. The median of these filtered mean depth values was used as a proxy for overall genome sequencing depth.

### RNA isolation

The total RNA was isolated from young leaves of plants growing in the greenhouse post-hardening using the RNeasy Plant Mini Kit (Qiagen) according to manufacturer’s instruction. RNA concentration and quality were determined using the DS-11 (DeNovix) spectrophotometer. Subsequently, samples were shipped for RNA sequencing.

### RNA sequencing

RNA from three independent transformed lines and one wild-type sample was sent for RNA sequencing to Novogene Europe (Munich). Sequencing reads were mapped against the construct gene sequences using BWA-MEM and further processed with SAMtools. Gene expression counts were normalized as tags per million (TPM) using the formula: (counts/length)/total number of reads x 106. All computations and visualizations were performed in R (https://cran.r-project.org/). The wild-type sample was used as a control to confirm the absence of transgene expression in non-transformed plants. High-throughput sequencing raw data have been deposited in the NCBI SRA under project number PRJNA1191157.

### Betalain extraction

Anthocyanins were extracted from leaves of plants growing *in vitro* according to the quantification method for the RUBY reporter (Liu J. et al., 2024). Absorbance was measured using the DS-11 (DeNovix) spectrophotometer.

## Results

### Establishing an *A*. *rhizogenes*-mediated genetic transformation system for *P. sieboldii*

*P. sieboldii* is easy to grow *in vitro*, and regeneration from roots is an efficient method for its micropropagation (Furuya and Hosoki, 2005). Somatic embryogenesis can be consistently induced from root segments cultured on a callus-inducing medium with a high concentration of auxin (NAA) under dark conditions. This promotes the formation of an embryogenic callus from which somatic embryos later develop (**Supplementary Figure S2A**). Subsequently, maturation, germination, and further growth of somatic embryos occur after subculturing onto hormone-free medium and exposure to light conditions (**Supplementary Figures S2B–G**). Root formation occurs without the supplementation of plant growth regulators, allowing for complete plantlet development (**Supplementary Figure S2H**).

Initially, we aimed to develop a transformation protocol for *P. sieboldii* using *A. tumefaciens*. In our transformation experiment with *A. tumefaciens*, somatic embryos expressing *RUBY* were formed, but whole-plant regeneration was not observed due to competition with non-transformed embryos and, primarily, low transformation efficiency (**Supplementary Figure S3**). The protocol for *A. tumefaciens*-mediated transformation is described in **Supplementary Method S1**.

**Figure 1:**
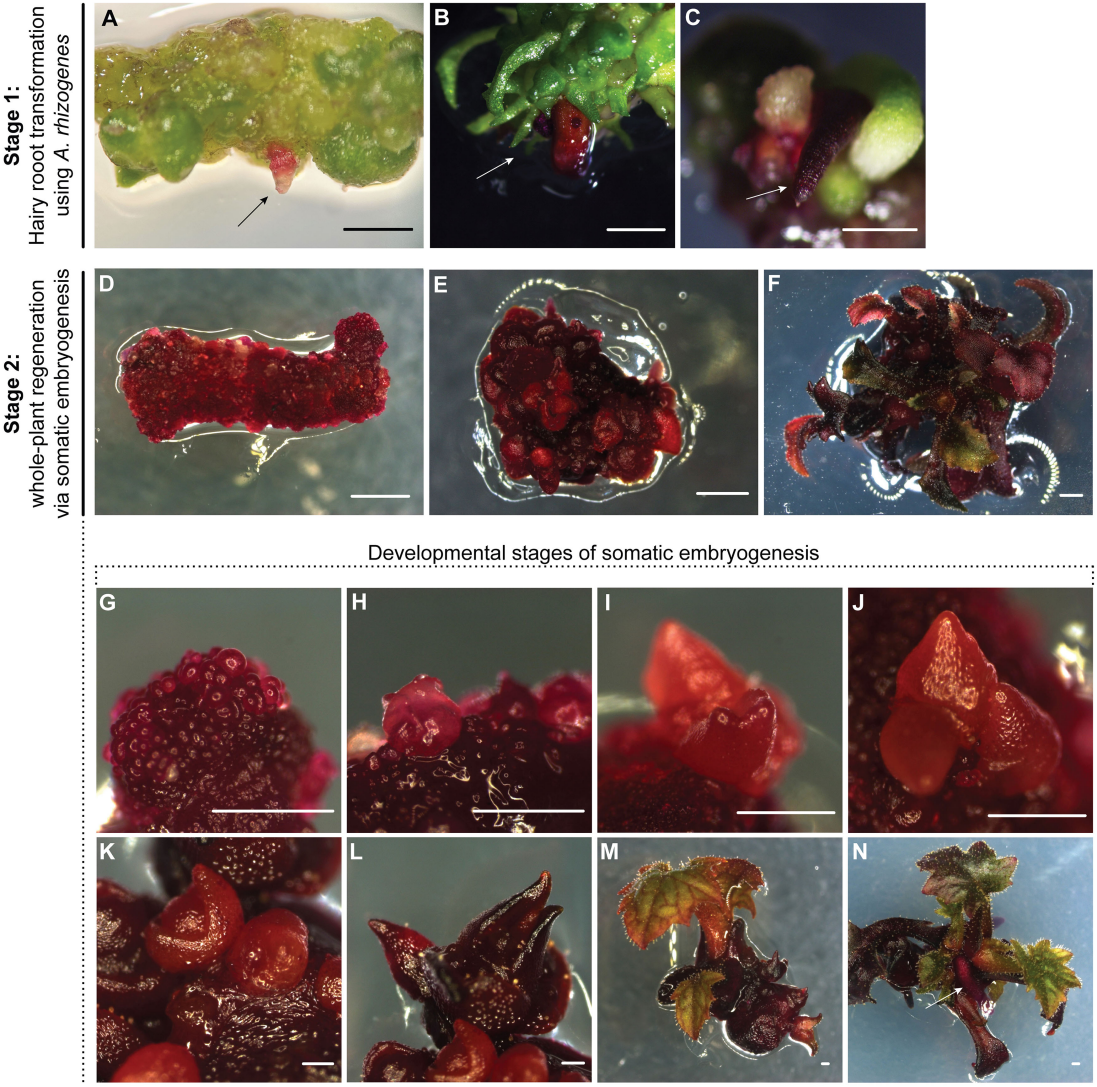
Hairy root transformation and whole-plant regeneration via somatic embryogenesis in *P. sieboldii*. **(A–C)** Stage 1: Hairy root transformation using *A. rhizogenes* strain A4. Transgenic hairy roots formed 4–6 weeks after inoculation and exhibited a strong red color due to the expression of the *RUBY* reporter. Arrows indicate transgenic hairy root formation. Scale bars = 1 mm. **(D–N)** Stage 2: Whole-plant regeneration via somatic embryogenesis. Explants derived from transgenic hairy roots were cultured on callus-inducing medium, leading to the formation of embryogenic callus and somatic embryos **(D)**, followed by embryo development and germination, ultimately resulting in regenerated plantlets **(E, F)**. **(G–N)** Developmental stages of somatic embryogenesis in *P. sieboldii*. Regeneration from transgenic hairy roots via somatic embryogenesis showing characteristic stages: globular (3 weeks, **G**), heart (4 weeks, **H**), torpedo (5 weeks, **I**), cotyledonary embryo (around 6 weeks, **J–L**), somatic embryo germination (2–4 weeks after transfer to MS20, **M**), and root development (4–6 weeks transfer to MS20, **N**). Arrows indicate root formation in plantlets. Scale bars = 1 mm.

Therefore, we decided to use a two-step approach, with the first step involving an initial hairy root transformation using *A. rhizogenes* A4 and subsequent whole-plant regeneration from transgenic hairy roots via somatic embryogenesis. The agropine-type RiA4 plasmid contains two independently transferred T-DNA fragments: TL-DNA and TR-DNA, which encode genes responsible for hairy root induction and agropine synthesis, respectively. However, only TL-DNA, particularly the *rolB* gene, is essential for hairy root induction (Bahramnejad et al., 2019). In the first step, root segments were pre-cultured on callus-inducing medium to form embryogenic callus, which is more susceptible to efficient transformation. Explants inoculated with *A. rhizogenes* developed transformed hairy roots after approximately six weeks (**Figures 1A-C**). The efficiency of transformed hairy root formation was 12%, with 52 out of 420 explants successfully forming hairy roots, 22 of which (42%) exhibited a vivid red color (**Table 2**). The overall hairy root transformation with efficiency with the binary vector containing the *RUBY* reporter was 5% (22/420). White roots resulted from the expression of the *rolB* gene from the TL-DNA of the RiA4 plasmid, while red roots were due to the co-transformation of genes from both RiA4 TL-DNA and the T-DNA of the 35S:RUBY binary vector. Additionally, transformed red shoots were regenerated through direct shoot organogenesis from callus, although this was a rare event, with only two independent transgenic lines (TG2 and TG8) being regenerated (0.5%) and, resulting in one group of regenerants (**Supplementary Figure S4**). Additionally, 10 plants were regenerated from transgenic hairy roots expressing *RUBY*, as described in the following section.

**Table 2:** P*. sieboldii* transformation efficiency using *A. rhizogenes*.

Number of explants inoculated with *A. rhizogenes*	Number of explants producing hairy roots	Number of explants producing white hairy roots	Number of explants producing red hairy roots	Number of plants regenerated through direct shoot organogenesis
420	52 (12%)	48* (11%)	22 (5%)	2 (0.5%)†

*The number of explants producing white hairy roots is not equal to the total number of explants producing hairy roots, since one explant can produce red and white hairy roots, only red, or only white.

†The number of plants regenerated through direct shoot organogenesis indicates plants regenerated directly from callus without the formation of hairy roots, followed by subsequent whole plant regeneration.

### Whole-plant regeneration from transgenic hairy roots

In the second step, whole plants were regenerated from transformed hairy roots. The 35S:RUBY plasmid contains a hygromycin resistance gene resistance gene for plant selection, however antibiotic selection was not applied during hairy root induction and whole-plant regeneration. Instead, transgenic hairy roots were identified based on the visible red pigmentation produced by the *RUBY* reporter. Only these hairy roots were selected for whole-plant regeneration, as they were assumed to contain T-DNA fragments from both the native RiA4 plasmid and the 35S:RUBY binary vector. Subsequently, red hairy roots were cut into small segments ~0.5–1 cm and placed on callus-inducing medium under dark conditions, which induced callus and somatic embryo formation after 4–6 weeks (**Figure 1D**). Embryogenic callus was induced on all explants derived from transformed hairy roots, with the average number of somatic embryos ranging from 10 to over 100 (**Table 3**). Subsequently, further embryo germination was observed following transfer to MS20 (**Figures 1E, F**). Typically, one root gave rise to many somatic embryos, which underwent characteristic developmental stages, including globular, heart-shaped, torpedo, and cotyledonary (**Figures 1G-N**). These embryos were easily separated from the callus and placed individually on fresh medium to facilitate their further development (**Figure 1M**). The resulting young plantlets developed roots without the need for additional plant hormones (**Figure 1N**). Early embryonic stages such as the globular stage were typically observed after 3–4 weeks of culture, followed by heart-shaped and torpedo stages at around 4–5 weeks. Cotyledonary stage embryos usually appear in 6 weeks. However, it is common to observe somatic embryos at different developmental stages simultaneously on the same explant. In total, we were able to regenerate 10 independent transgenic lines derived from transgenic hairy roots carrying *RUBY*.

**Table 3:** Somatic embryogenesis efficiency of *P. sieboldii* from transgenic hariy roots.

Number of red hairy roots	Callus induction rate	Somatic embryo formation rate	Typical range (embryos per explant)	Number of independent transgenic lines	Number of regenerated plants per transgenic line
10*	10 (100%)	10 (100%)	10-100+	10	≤ 30

*Each red hairy root corresponds to an independent transformation event.

**Figure 2:**
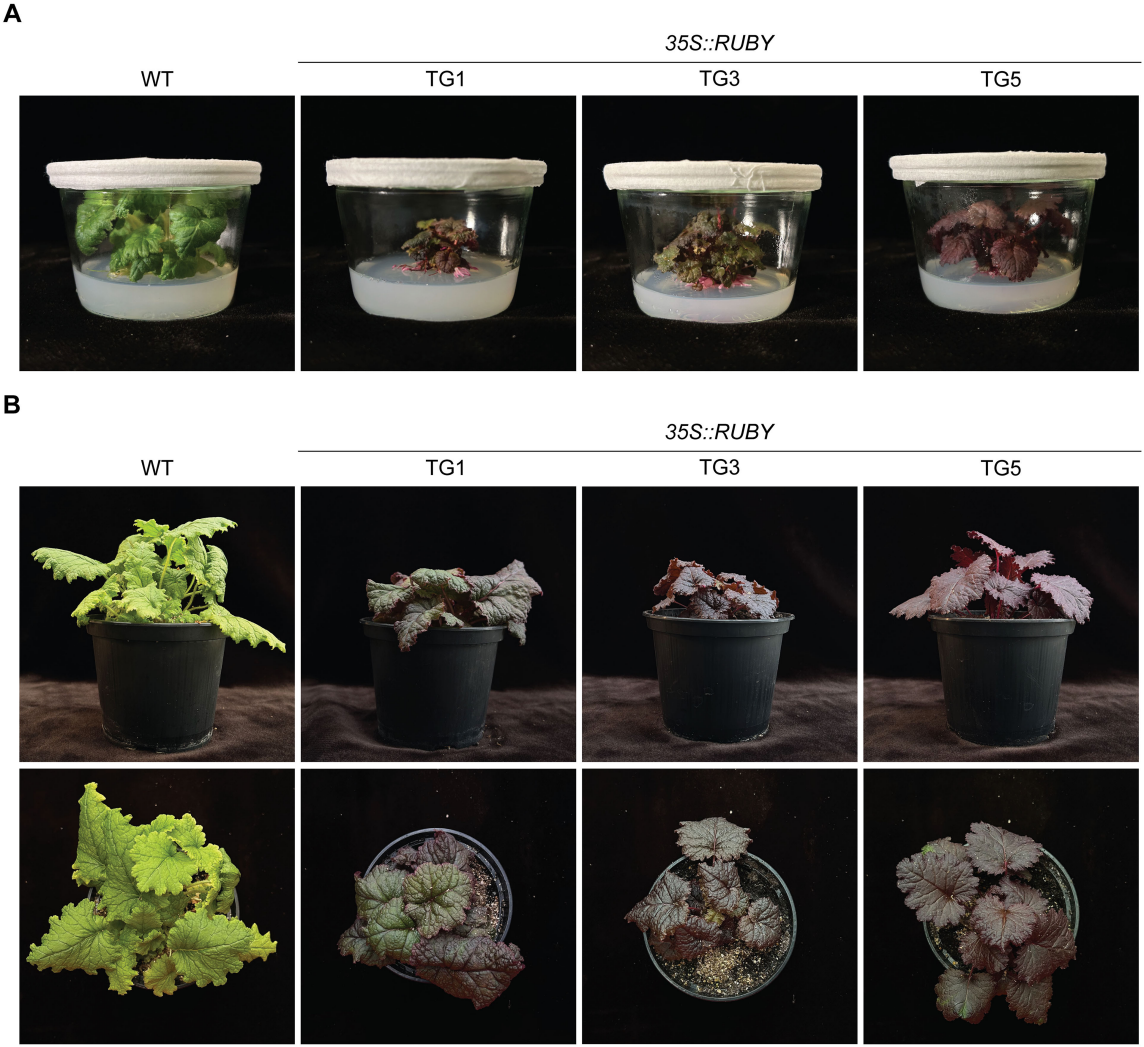
Acclimatization of regenerated *P. sieboldii* from transformed hairy roots induced by *A*. *rhizogenes*. **(A)** *P. sieboldii* plants cultivated in sterile jars, ready to be transferred to soil. **(B)** Acclimatization of transgenic *P. sieboldii* to greenhouse conditions. WT, wild-type *P. sieboldii*; TG, transgenic lines transformed with 35S:RUBY.

Plantlets were transferred to sterile glass jars for 9 weeks to allow further development of the root system before being transferred to soil. All plants transferred to the greenhouse survived acclimatization, and three independent transgenic lines (TG1, TG3, and TG5) were selected for further detailed molecular analysis, such as RNA-seq and whole-genome sequencing (**Figures 2A, B**). In total, 12 independent transgenic lines were established as a result of separate transformation events. The regenerated plants exhibited strong red coloration in all tissues. However, one transgenic line (TG6), regenerated from a transformed red root, showed strong *RUBY* expression only in the roots, with the aerial tissues remaining mostly green (**Supplementary Figure S5**). This method enables efficient regeneration of *P. sieboldii* from transgenic hairy roots; in our experiment, we successfully regenerated up to 30 transgenic plantlets from a single transgenic line, which were then acclimatized to greenhouse conditions.

### Molecular analysis of transformed plants

**Figure 3:**
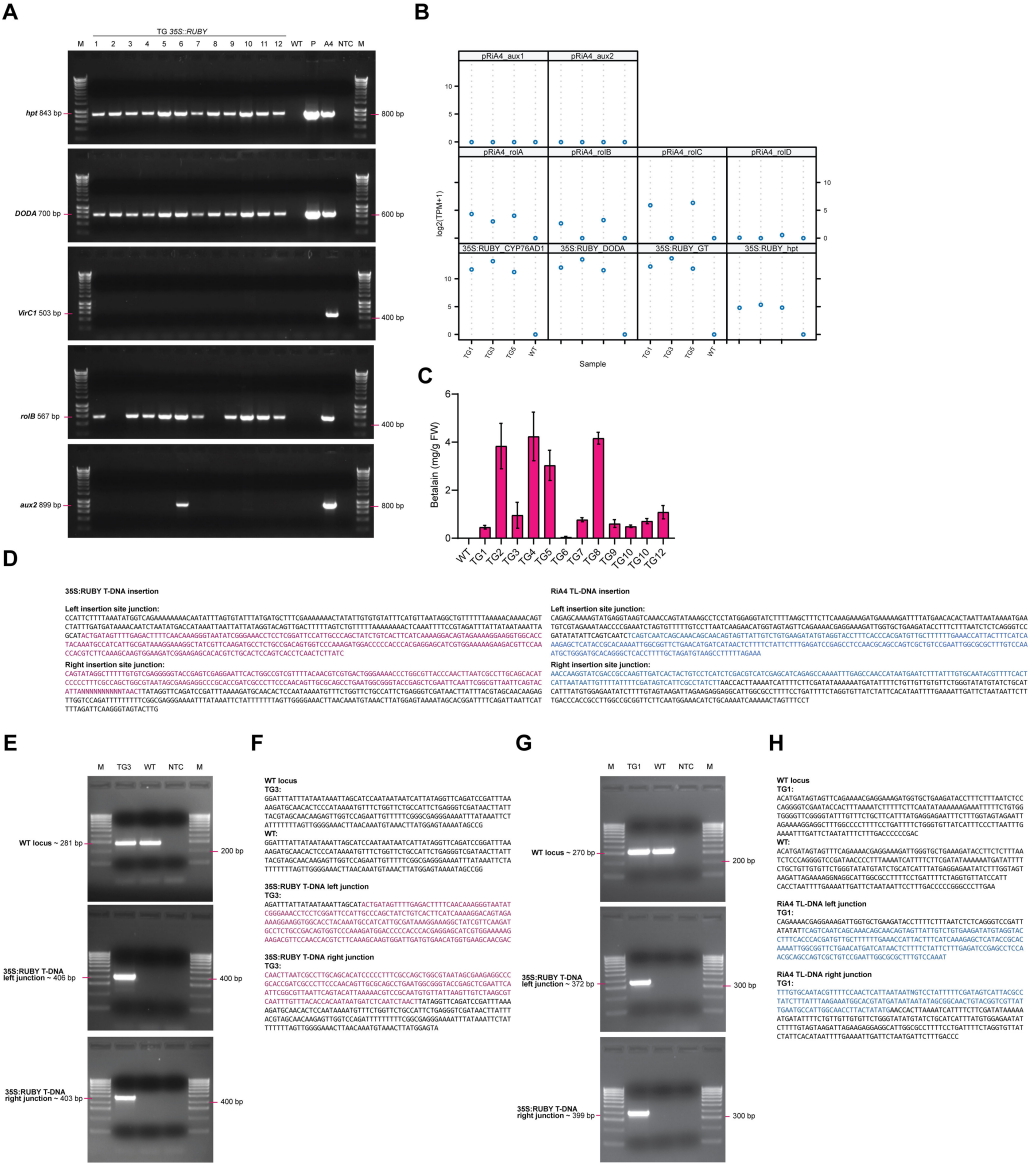
Molecular analysis of transgenic *P. sieboldii*. **(A)** PCR detection of transgenes in transformed *P. sieboldii*. M: marker, TG1–TG12: independent transgenic lines, WT: wild-type *P. sieboldii*, P: 35S:RUBY plasmid, A4: *A. rhizogenes* A4 used for the transformation experiment, NTC: no template control. Analyzed genes: *hpt*, *DODA*, *rolB*, *VirC1*, *aux2*. **(B)** Gene expression analysis of transgenic *P. sieboldii* via RNA-seq. Genes from the 35S:RUBY plasmid: *CYP76AD*, *DODA*, *GT*; TL-DNA (*rolA*, *rolB*, *rolC*, *rolD*); TR-DNA (*aux1*, *aux2*) of pRiA4. **(C)** Quantification of betalain concentration in the leaves of transgenic *P. sieboldii*. TG: independent transgenic lines, WT: wild-type *P. sieboldii*, n=3. **(D)** T-DNA insertion sites predicted from DNA-seq data. Sequences from the 35S:RUBY plasmid are in magenta, and those from the RiA4 plasmid are in blue. Insertions were identified in TG3 (35S:RUBY) and TG1 (RiA4). **(E, G)** PCR amplification of the left and right T-DNA insertion junctions for RUBY and RiA4, respectively. M, marker; TG, transgenic line; WT, wild-type *P. sieboldii*; NTC, no template control. **(F, H)** Sanger sequencing validation of the T-DNA insertion sites.

To confirm the successful integration and expression of the transgenes introduced by *A. rhizogenes*, a series of molecular analyses were carried out. Only one plant per independent transgenic hairy root was used, since plants derived from the same root are likely clonal; as such only plants derived from different original root explants were considered as independent transformation events. PCR was performed to verify the presence of transgenes in the genomic DNA of transgenic plants. The integration of genes from the 35S:RUBY construct, specifically *hpt*, and *DODA*, was confirmed in all transgenic lines. No *A. rhizogenes* contamination was detected, using *VirC1* as a control. Plants regenerated from transgenic roots (TG: 1, 3, 4, 5, 6, 7, 9, 10, 11, 12) exhibited integration of the *rolB* gene derived from TL-DNA of the Ri plasmid. Only one line (TG6) showed integration of TR-DNA from the Ri plasmid, where the *aux2* gene was amplified. In contrast, TL-DNA genes were not detected in plants regenerated via direct shoot organogenesis in lines TG2 and TG8. Additionally, no transgenes were amplified from wild-type *P. sieboldii* (**Figure 3A**).

To determine the expression levels of transgenes introduced by *A. rhizogenes* in plants regenerated from transformed hairy roots, the expression of *CYP76AD*, *DODA*, *GT*, and *hpt* derived from the 35S:RUBY plasmid was analyzed in three selected transgenic lines (TG1, TG3, and TG5) using RNA-seq on leaf samples. High levels of transgene expression were observed in these transgenic plants. The expression of genes derived from the TL-DNA of the Ri plasmid (*rolA*, *rolB*, *rolC*, *rolD*) varied among the transgenic lines, while no expression of genes from the TR-DNA (*aux1*, *aux2*) was detected, indicating a lack of TR-DNA integration consistent with the PCR results. In contrast, the control wild-type plant showed no detectable expression of any transgene (**Figure 3B**).

The betalain concentration in the leaves of transgenic lines was quantified using spectrophotometric analysis. Betalain was detected in all transgenic lines, while it was absent in wild-type *P. sieboldii*. Among the transgenic lines, betalain levels varied significantly, ranging from 0.05 mg/g FW to 4.24 mg/g FW. The average concentration across all transgenic lines was 1.70 mg/g FW (**Figure 3C**).

T-DNA insertion sites in transgenic plants were determined by whole-genome sequencing in selected two transgenic lines (**Figure 3D**, **Supplementary File S2**). Flanking sequences were amplified by PCR and confirmed through Sanger sequencing. T-DNA sequence containing the *RUBY* reporter gene was identified and validated in TG3 (**Figures 3E, F**), while TL-DNA derived from the RiA4 plasmid was detected and confirmed in transgenic line TG1 (**Figures 3G, H**). These results confirm the successful expression and integration of transgenes responsible for betalain biosynthesis in transgenic plants.

To estimate the number of T-DNA insertions in transgenic lines, we utilized whole-genome sequencing data.We used conserved single-copy genes from *P. veris* to estimate genome coverage in our whole-genome sequencing data of the TG1, TG3, and TG5 transformants. The median meandepth values, computed using samtools coverage, were used as proxies for overall genome coverage and were 3.8, 5.2, and 4.4 for TG1, TG3, and TG5, respectively. The corresponding mean depth values for the inserted RUBY T-DNA region were 4.3, 4.1, and 3.0. Based on the ratio between the coverage estimates for the T-DNA region and the genome as a whole, we concluded that TG3 and TG5 most likely harbor a single heterozygous T-DNA insertion (**Supplementary Table S1**). In contrast, in TG1, where the T-DNA coverage slightly exceeds the genome coverage (ratio > 1), there appear to be either two heterozygous T-DNA insertions or a tandem insertion at one locus. However, due to the relatively low sequencing depth, only a single T-DNA insertion junction could be confidently resolved.

### Stable germline transmission of transgenes

**Figure 4:**
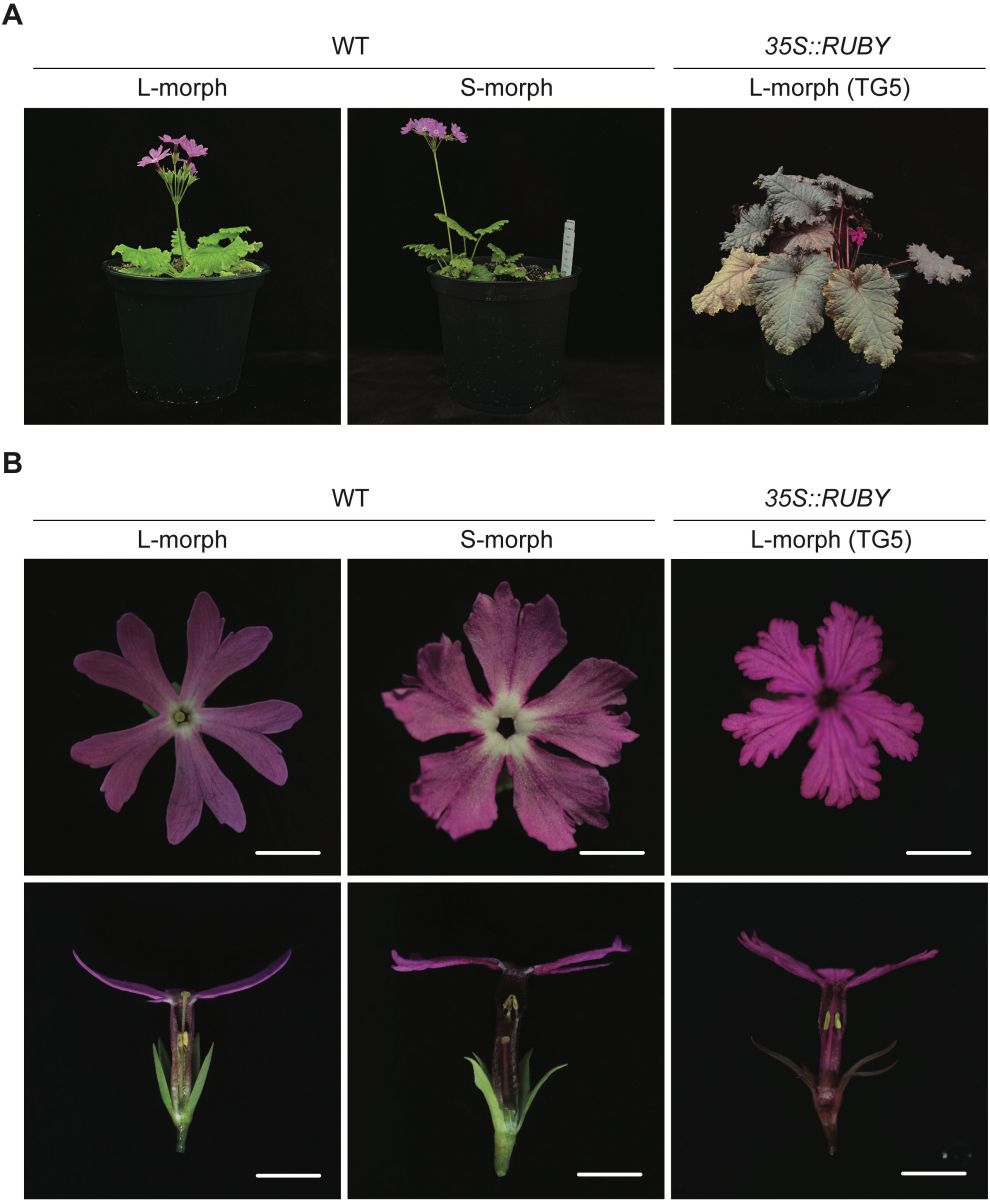
Flowering of transgenic *P. sieboldii*. **(A)** *P. sieboldii* flowering five months after transfer to greenhouse conditions for five months. **(B)** Flower morphology of *P. sieboldii*. WT, wild-type *P. sieboldii* (L-morph and S-morph), TG, transgenic line. Scale bars = 5 mm.

**Figure 5:**
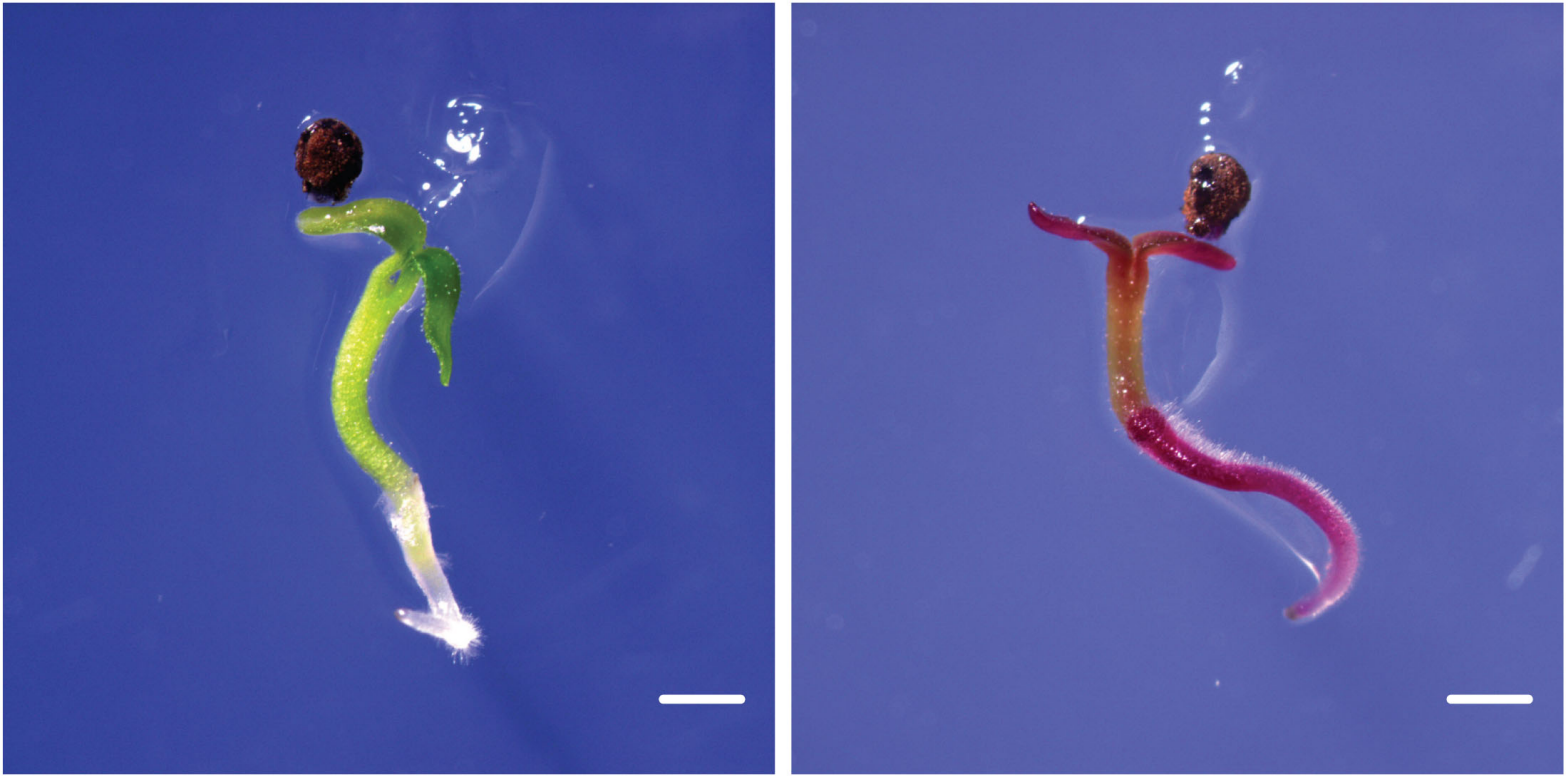
Stable germline transmission of transgenes. Transgene segregation in *P. sieboldii* T1 seedlings germinated *in vitro*. The non-transgenic seedling, which is green, is on the left, while the transgenic seedling expressing the *RUBY* reporter is on the right, showing betalain accumulation in all tissues. Scale bars = 1 mm.

*P. sieboldii* exhibits a heteromorphic self-incompatibility system, preventing self-pollination and subsequent seed formation. Therefore, reciprocal crosses between L- and S-morph plants are necessary to confirm transgene inheritance. Although *P. sieboldii* is a perennial plant and does not always flower in its first year of cultivation due to the need for vernalization, in our case, we observed flowering. One transgenic line (TG5) first flowered five months after being transferred to the greenhouse (**Figures 4A, B**). To confirm transgene transmission to the next generation, T0 plants were crossed with wild-type *P. sieboldii*. TG5 (L-morph) was used as the pollen recipient in crosses with wild-type S-morph plants. Following hand pollination, seed capsules began to swell and were harvested after approximately six weeks. The seeds were then sown and germinated *in vitro* (**Figure 5**). Among the germinated seeds, 33% (9/27) successfully sprouted, and 55% (5/9) exhibited *RUBY* expression, confirming stable transgene transmission to progeny via gametes. Furthermore, the observed Mendelian inheritance ratio of 1:1 suggests a single heterozygous T-DNA insertion, which is fully consistent with the whole-genome sequencing data.

T1 plants showed variation in growth rate and size both *in vitro* and after transfer to the greenhouse (**Supplementary Figure S6**). This variation may be caused by transgene expression; however, since the T1 plants originated from the cross between the transformed plant and the wild-type parent and remain heterozygous for the transgene, it is difficult to directly attribute the observed phenotypes to the transgene.

## Discussion

Here, we developed a protocol for the genetic transformation of *P. sieboldii* using *A. rhizogenes*, followed by whole-plant regeneration from transgenic hairy roots via somatic embryogenesis. The availability of an efficient and reproducible regeneration method is a crucial factor for the successful establishment of transformation systems, as it directly impacts the ability to generate stable transgenic lines, especially in recalcitrant species (Bélanger et al., 2024). Only a limited number of plants can be transformed by methods such as the floral dip (Clough and Bent, 1998), *de novo* induction of meristems (Maher et al., 2020), or the cut-dip-budding method (Cao et al., 2023) which can omit the need for regeneration through tissue culture. In most cases, however, *in vitro* techniques are necessary for the generation of transformed plants.

Recalcitrant species are resistant to successful genetic transformation, which is often caused by high sensitivity to *Agrobacterium*, which limits the effectiveness of common transformation methods (Pitzschke, 2013). Despite these challenges, *P. sieboldii* and other recalcitrant plants are highly valuable because they possess unique traits not found in widely studied model organisms such as *Arabidopsis* or tobacco (Levengood et al., 2024). These distinctive characteristics make them desirable targets for transformation (Cardi et al., 2023). Recent advances in plant tissue regeneration and stable transformation protocols for recalcitrant species have demonstrated that successful genetic modification is achievable through optimized methodologies (Zhang et al., 2024).

Our preliminary results demonstrated the feasibility of establishing a transformation protocol for *P. sieboldii* using *A. tumefaciens*. This notion is supported by our observation of direct transgenic shoot organogenesis during transformation with *A. rhizogenes*. However, efficient plant regeneration remains a challenge. Thus, further optimization of the *A. tumefaciens*-mediated transformation system is necessary and should focus on improving transformation efficiency and enhancing the regeneration of plants from transformed tissue.

**Figure 6:**
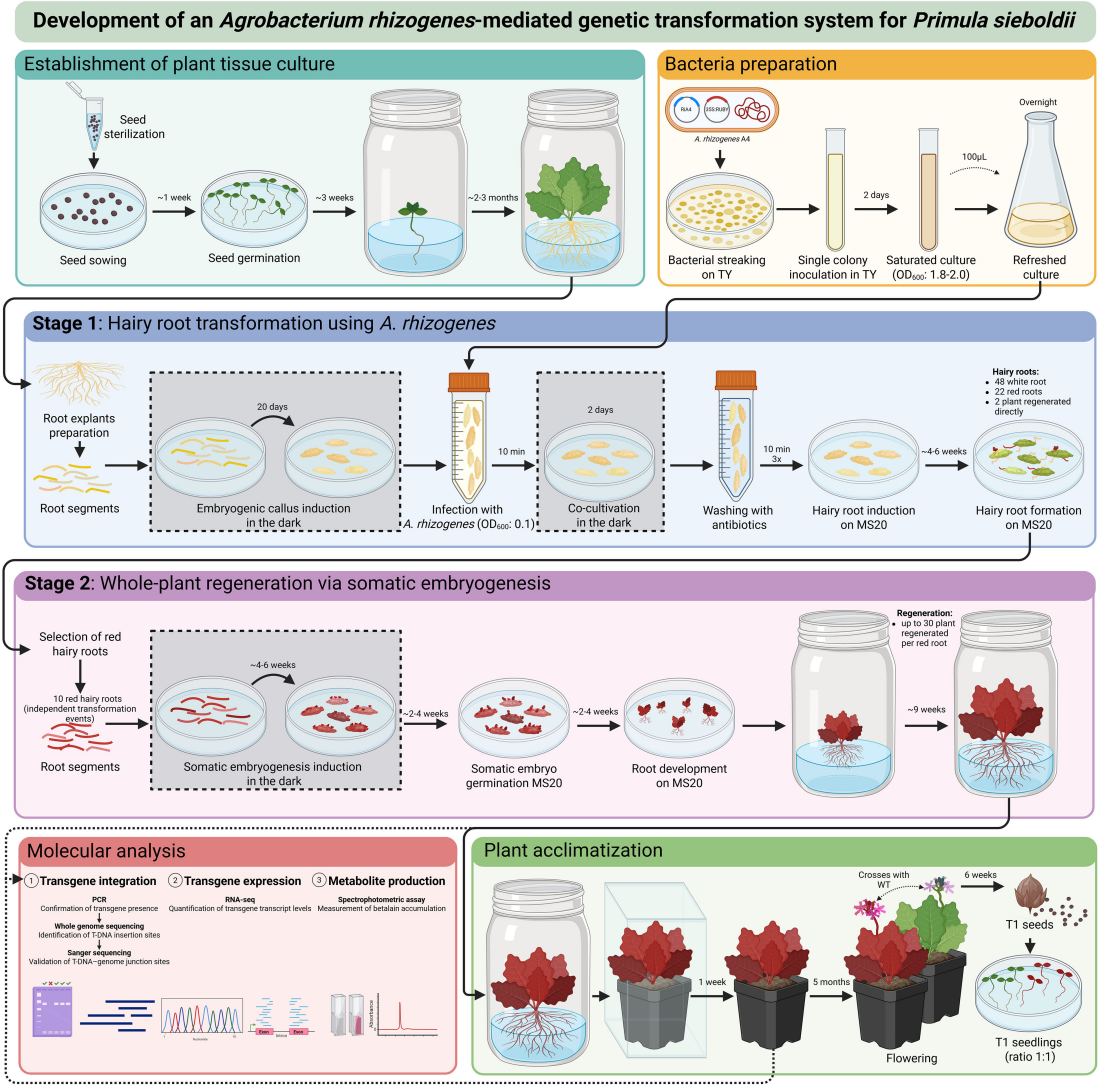
Schematic overview of the *A. rhizogenes*-mediated transformation protocol for *P. sieboldii*. The diagram illustrates explant preparation and bacterial culture, followed by genetic transformation using *A. rhizogenes*. In the first stage, transgenic hairy roots are induced; in the second stage, whole plants are regenerated through somatic embryogenesis. The workflow also includes plant acclimatization to greenhouse conditions and molecular analysis to confirm successful transformation.

In contrast, *A. rhizogenes*-mediated transformation was successfully established. We were able to induce hairy root formation, followed by whole-plant regeneration via somatic embryogenesis. A schematic summary of the transformation method, along with its time frame, is shown in **Figure 6**. The hairy root transformation and whole-plant regeneration phases combined are estimated to take approximately 6 to 8 months, while acclimation and flowering require about additional 5 months. Development of a transformation system based on *A. rhizogenes* is beneficial and essential for these species in which transformation using *A. tumefaciens* is ineffective and results in low transformation efficiency, while regeneration from the transformed hairy root is successful (Liu L. et al., 2024). Transgenic hairy roots are not prone to forming chimeric plants as they are initiated from a single transformed cell, making them ideal explants for generating non-chimeric transgenic calli for subsequent whole-plant regeneration (Xu et al., 2020).

The initial hairy root transformation experiment was performed to assess whether *A. rhizogenes* A4 could induce transgenic hairy roots expressing the *RUBY* reporter in *P. sieboldii*, which was indeed observed. In this study, we present the second attempt, detailing the number of explants and regenerated plants obtained. In a third experiment, we followed the described protocol to induce mutations using CRISPR-Cas9, generating eight independent transgenic Ri lines regenerated from transformed hairy roots. Among these, one line carried the transgene from the CRISPR-Cas9 vector, but the desired mutation was not induced (**Supplementary Figure S7**, **Supplementary Method S2**).

Due to its regenerative properties, embryonic callus is one of the most suitable tissues for genetic transformation. Various methods of inducing somatic embryogenesis in different plant species have been described, with induction primarily depending on the specific combinations and concentrations of plant hormones in the medium. In *Primula*, successful somatic embryogenesis has been reported in only a few species. In *P. cuneifolia* Ledeb. var. *hakusanensis*, somatic embryogenesis was achieved using leaf-derived explants cultured on medium supplemented with TDZ or zeatin (Shimada et al., 1997). *P. nutans* Georgi, *P. purdomii* Craib, and *P. stenocalyx* Maxim. were induced to form somatic embryos using TDZ and NAA (Otani et al., 1999). Similarly, high concentrations of auxins have been shown to effectively stimulate somatic embryogenesis in root-derived explants, including transgenic hairy roots induced by *A. rhizogenes* in *P. sieboldii*.

Typically, reporter genes are used to visualize gene expression, and several systems were set up to be used in plant transformation such as *luciferase* (*luc*) (Millar et al., 1992), *β-glucuronidase* (*uidA*) (Jefferson et al., 1987), or *green fluorescent protein* (*gfp*) (Sheen et al., 1995) and its variants. In our study, the *RUBY* reporter enabled easy differentiation of transformation events based on the production of red pigment by transformed cells. *RUBY* offers advantages over other reporters as it does not require special equipment or chemical treatment (He et al., 2020). The strong transgene expression observed during the early stages of transformation, as well as in mature transgenic plants demonstrated its suitability as a reporter gene in *P. sieboldii*.

Selective agents are commonly used to inhibit the growth of non-transformed cells and typically rely on genes conferring resistance to selective agents like antibiotics or herbicides. In our study, it was not tested if the implementation of antibiotic selection during the early stages of transformed hairy root formation could inhibit the regeneration of non-transformed cells. However, this approach could potentially enhance the co-transformation efficiency of the two constructs used. This is because TL-DNA from the Ri plasmid and T-DNA from the binary vector integrate independently at different sites in the plant genome, necessitating selection for both transformation events. Hairy root development would occur only if TL-DNA from the Ri plasmid is integrated into the plant genome, while hygromycin resistance would be conferred by the *hpt* gene from the 35S:RUBY plasmid. Selection based on red pigment production by transgenic *P. sieboldii* was straightforward, whereas antibiotic selection will likely require concentration optimization; however, our protocol provides a robust basis for this. Furthermore, achieving visual selection without antibiotics enables the development of a highly efficient screening system for transformants and is applicable to a wide range of plant species (Dutt et al., 2018; Saika and Toki, 2009; Lin et al., 2011).

Transformation and whole-plant regeneration using *A. rhizogenes* often result in altered plant morphology due to the expression of oncogenes from the Ri plasmid (Altamura, 2004). Typical phenotypic changes include wrinkled leaves, shortened internodes, and reduced apical dominance (Tepfer, 1984; Desmet et al., 2021). However, *P. sieboldii* plants regenerated from transgenic hairy roots did not show similar gross morphological changes in their phenotype.

Additionally, flower morphology, including pollen viability of transgenic plants, may be affected by genetic transformation using *A. rhizogenes* (van Altvorst et al., 1992). In species where self-pollination is not feasible or where strong self-incompatibility prevents it, transformed plants can serve as pollen recipients to facilitate transgene transmission to progeny. In our study, transgenic *P. sieboldii* plants were used as pollen recipients in legitimate crosses, resulting in a full set of viable seeds. Furthermore, transgene inheritance in the next generation has been demonstrated in other species and our findings support the feasibility of achieving similar outcomes in *P. sieboldii* (Crane et al., 2006; Desmet et al., 2020).

This study presents the first genetic transformation method for *P. sieboldii*, providing a reliable protocol for genetic engineering of this species using *A. rhizogenes*. The establishment of the transformation method can advance our understanding of genetic and molecular control of self-incompatibility and heterostyly in *Primula* but also offers practical applications for plant biotechnology.

## Data Availability

The datasets presented in this study can be found in online repositories. The names of the repository/repositories and accession number(s) can be found below: https://www.ncbi.nlm.nih.gov/, PRJNA1191157.
