# Supplementary material for: Genetic transformation of Primula sieboldii using Agrobacterium rhizogenes and whole-plant regeneration from transgenic hairy roots
Source: Front Plant Sci. 2025 Jul 25;16:1623387. doi: 10.3389/fpls.2025.1623387 (PMC12331739; doi:10.3389/fpls.2025.1623387)
Supplement: Supplementary file 1 [file DataSheet1.docx]

Supplementary Material

# Supplementary Data

**Supplementary File S1.** Oligonucleotide sequences

Molecular analysis of transformed plants:

FP hpt, ATTTGTGTACGCCCGACAGT

RP hpt, CTCGGAGGGCGAAGAATCTC

FP DODA, CCGTCGAGGATACACATCCG

RP DODA, TCCCAGGAGGAGTGGATCAG

RP VirC1, CGCGATCCTGAGATTCCGTT

RP VirC1, GGAAAGAAAACGCCCTACGC

FP rolB, GAGAGTCGCAGGGTTAGGTC

RP rolB, GAAACGATGGGCTCTTGCAG

FP aux2, AAGTAACTGTGGTAGGCCGC

RP aux2, CTAGCTGGCGTTCCCCTATG

Amplification of the 35S:RUBY T-DNA insertion site:

FP WT locus, AGGTACAGTTGACTTTTTAGTCTGT

RP WT locus, TGAAAATCCGTGCTATTTTACTCCA

RP Left insertion site junction, TCACTGGCCGTCGTTTTACA

FP Right insertion site junction, GCCCTTTGGTCTTCTGAGACT

Amplification of the RiA4 TL-DNA insertion:

FP WT locus, TGAATGTCGTAGAAATAACCCCGA

RP WT locus, CAAGGCGGTGGGTCAAAGAA

RP Left insertion site junction, GTGCATCCCAGCATTTGGAC

FP Right insertion site junction, CATCGAGCATCAGAGCCAAAAT

**Supplementary File S2. Sequence transitions for T-DNA insertions** 
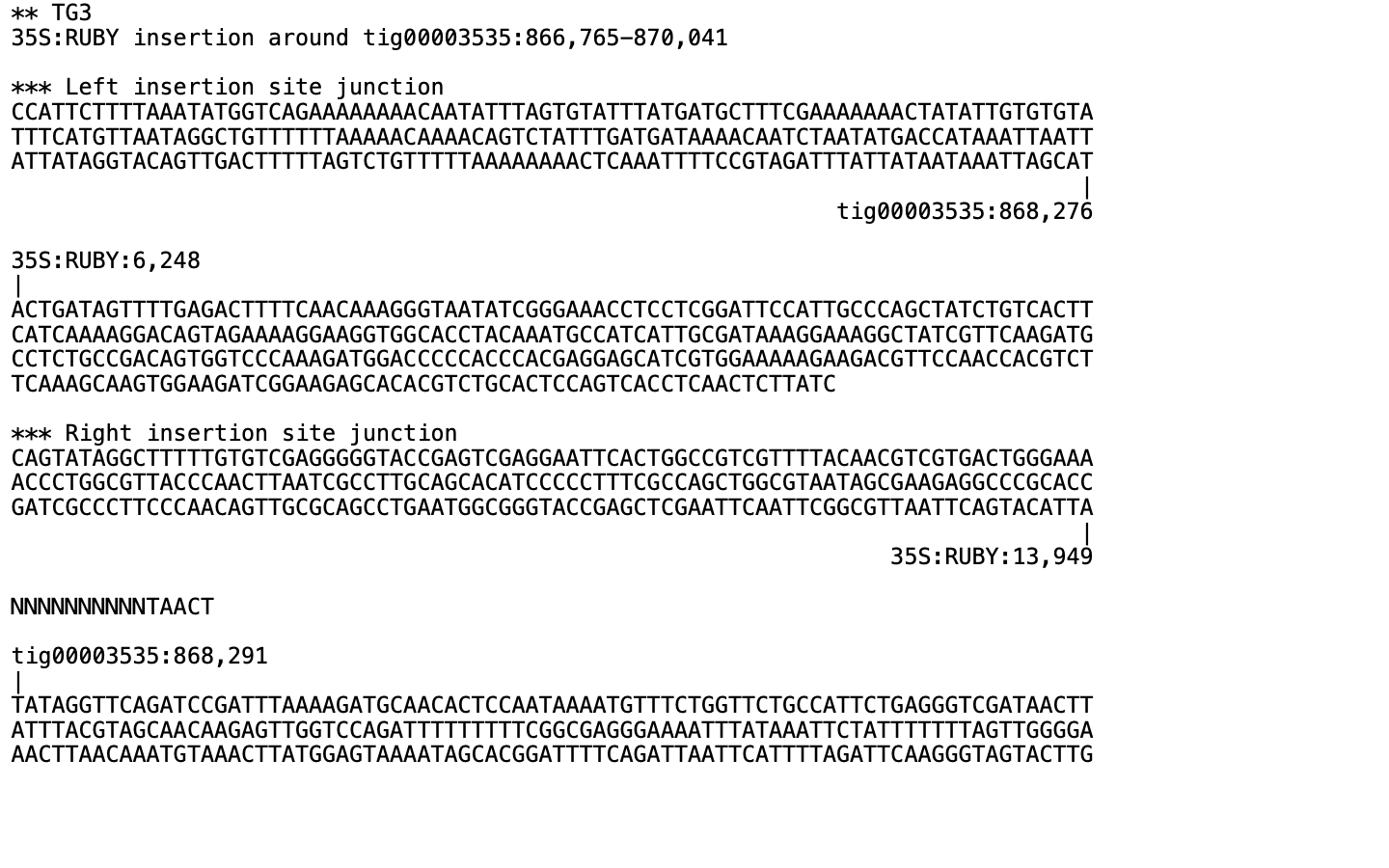

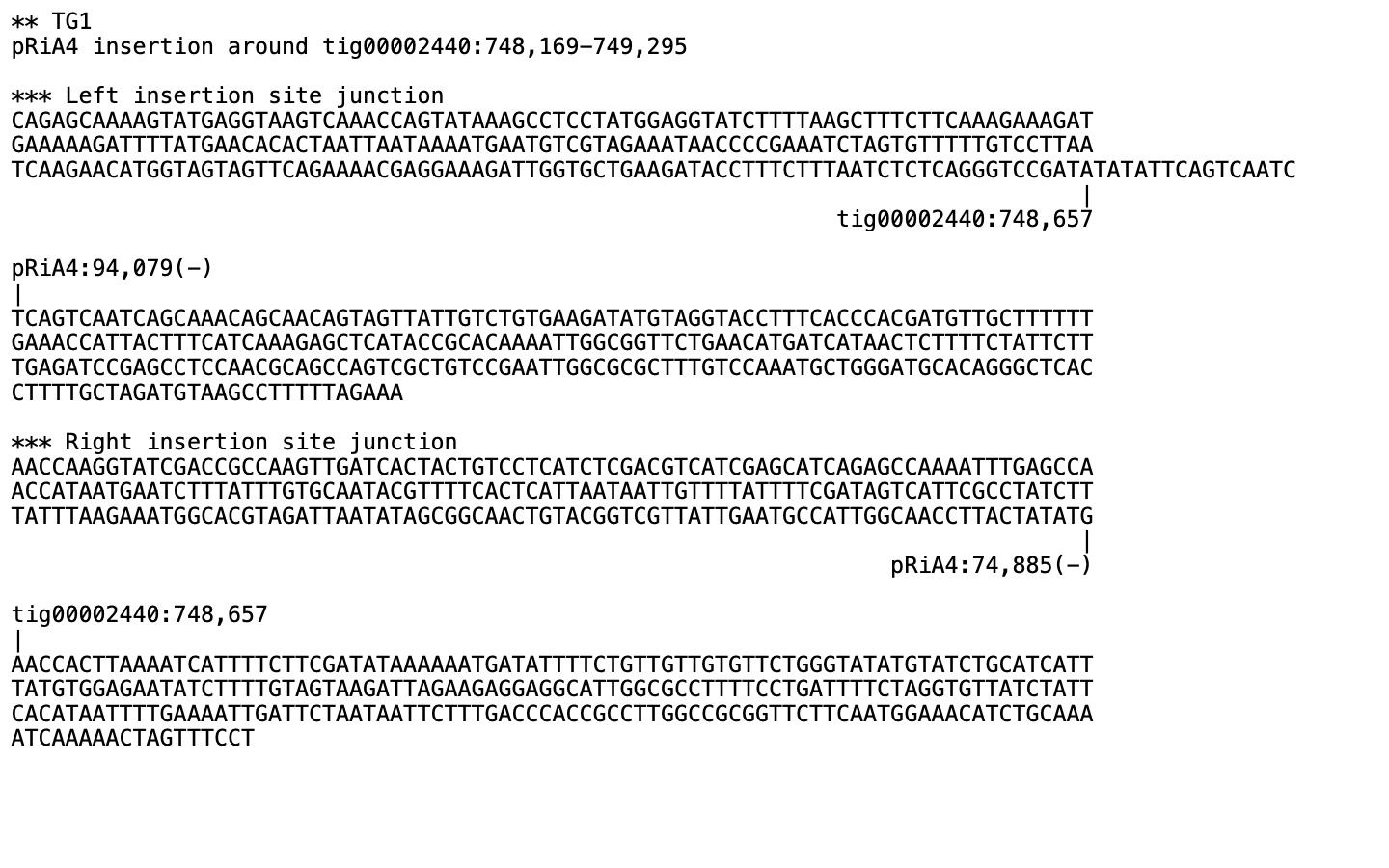


**Supplementary Method S1**

**Establishing an *A. tumafaceins*-mediated genetic transformation system for *P. sieboldii***

*Agrobacterium tumefaciens* strain GV3101 (GOLDBIO, Cat#CC-207-TR) was transformed with the 35S:RUBY construct following the method described for *A. rhizogenes*. Transformed bacteria were plated on LB medium supplemented with 50 mg/L spectinomycin, 50 mg/L rifampicin, and 100 mg/L gentamicin, and incubated at 28°C for 2 days. A single colony was then inoculated into 5 mL of LB medium containing the same antibiotics and cultured at 28°C shaking at 220 rpm for 2 days. Subsequently, 10 μL of this culture was transferred into 100 mL of fresh LB medium supplemented with the same antibiotics one day prior to transformation and incubated overnight under the same conditions. The 100 mL bacterial culture was centrifuged at 6,000 rpm for 15 minutes. The supernatant was discarded, and the bacterial pellet was resuspended in MS20 medium. The OD_600_ was measured using a BioPhotometer (Eppendorf) and adjusted to 0.1. Acetosyringone was then added to the suspension at a final concentration of 200 μM. Explants were prepared following the same procedure as for *A. rhizogenes*. Embryogenic callus tissue was used for transformation, and co-cultivation was carried out for 2 days. Then, the explants were washed with antibiotics as described for *A. rhizogenes*, and transferred to callus induction medium until somatic embryo formation was observed.

**Supplementary Method S2**

**Cloning strategy for a CRISPR-Cas9 vector**

To amplify Cas9-TLS2, PCR was performed using CloneAmp HiFi PCR Premix (Takara) with the following primers (Cas9_for_SpeI GAGAGGACTAGTTGAAGCTAGTCGACTCTAGCCTAGAATGGATTAC, Cas9_rev_HindIII CGAAAGCTTAATTAAGAATTATCAACCACTTTGTACAAGAAAGCTG). The Cas9-TLS2 plasmid (Addgene #196978) was used as the template. The PCR product was digested with SpeI and HindIII and purified using the NucleoSpin Gel and PCR Clean-up Kit (Macherey-Nagel). Subsequently, the 35S:RUBY vector was also digested with SpeI and HindIII and gel purified. The insert and backbone were then ligated, and the resulting plasmid was named pKG1. The plasmid was verified by Sanger sequencing.

To amplify the sgRNA scaffold with TLS2 and the *KFB^T^* sgRNAs using the following primers (KFB_sgRNA1_for CGCGGTCTCCATTGCAGGTTCCTAAGTACCCAGGGTTTTAGAGCTAGAAATAGCAAGTTAAAATAAGGCTAGTCCGTTATCAACTGAAAAAGT, KFB_sgRNA2_rev GCGGGTCTCTAAACTCCCGCGATGTATACATGTCAATCTCTTAGTCGACTCTACCAATATATAAACAGAGCTTCTATTTTCAACTGAACGATG), using plasmid gRNA NIA TLS2 (#196981) as template. These fragments were ligated via a Golden Gate reaction into plasmid Fragment 1 TLS2 (#196983) and named pKG2. The plasmid integrity was verified by Sanger sequencing.

The 2xsgRNA cassette from pKG2 was amplified using the following primers (2xsgRNA_AscI_for AAAGGCGCGCCCGACTTGCCTTCCGCACAATACATCATTTC, 2xsgRNA_PacI_rev CCCTTAATTAACCAAGCGAGCAAAAGCAGGTGCGCTGCAGTATTGG). The PCR product was digested with AscI and PacI and purified using a column-based method. The plasmid pKG1 was also digested with AscI and PacI, and gel purified. The insert and backbone were then ligated, and the resulting plasmid was named pKG3, which was verified by Sanger sequencing. Subsequently, pKG3, a CRISPR-Cas9-based vector, was used for the *P. sieboldii* transformation. The plasmids used in this study were originally gifts from Friedrich Kragler. They are available from Addgene, and the cloning strategy followed previously described methods (Yang et al., 2023).

## Supplementary Figures


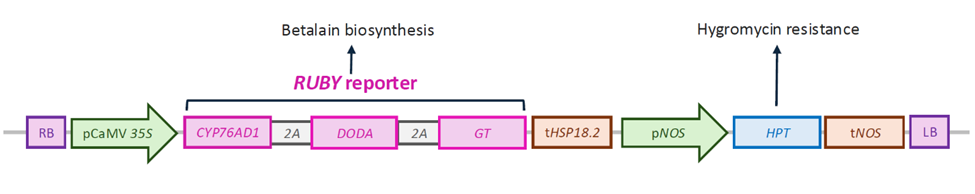


**Supplementary Figure ~~S2~~ S1.** T-DNA fragment of the 35S:RUBY binary vector. The 35S:RUBY plasmid is a binary vector containing the reporter gene *RUBY*, which encodes enzymes involved in the betalain biosynthetic pathway. These enzymes convert tyrosine into vividly red betalain. In addition, this vector includes the hpt gene, which confers hygromycin resistance. RB: right border; pCaMV *35S* (Cauliflower Mosaic Virus promoter); *CYP76AD* (*P450 oxygenase*); *DODA* (*L-DOPA 4,5-dioxygenase*); *GT* (*glucosyltransferase*); *2A* peptides; t*HSP18.2* terminator; p*NOS* (nopaline synthase promoter); *hpt* (hygromycin phosphotransferase); t*NOS* (nopaline synthase terminator); LB: left border.

**
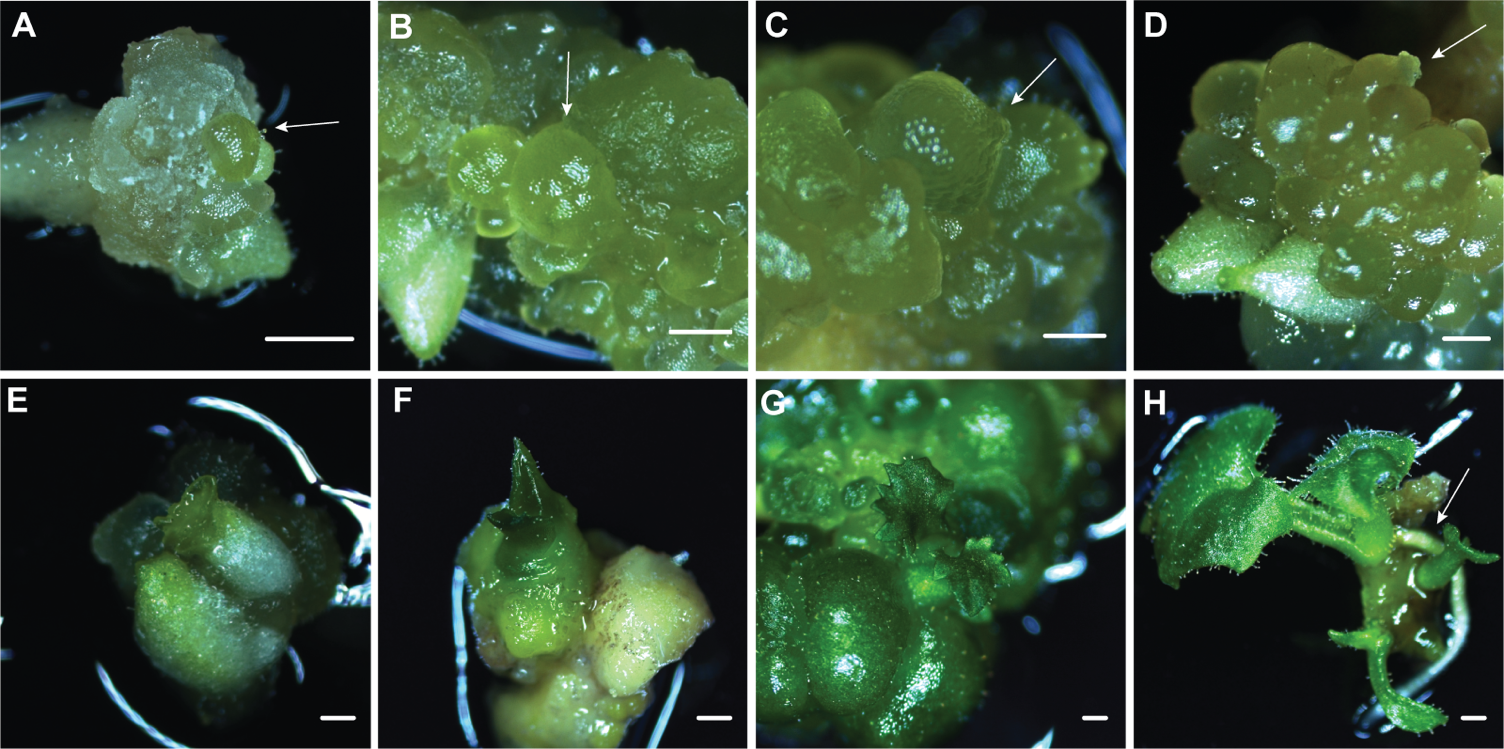
**

**Supplementary Figure ~~S1~~ S2.** Whole-plant regeneration from root segments via somatic embryogenesis in *P. sieboldii*. Formation and development of somatic embryos through different stages, including the globular at 3 weeks (**A**), heart at 4 weeks (**B, C**), torpedo at 5 weeks (**D**), and cotyledonary around 6 weeks (**E**) stages, followed by somatic embryo germination (**F, G**) and root development (**H**). Arrows indicate somatic embryos at different developmental stages and root formation in the plantlet. Scale bars = 1 mm.


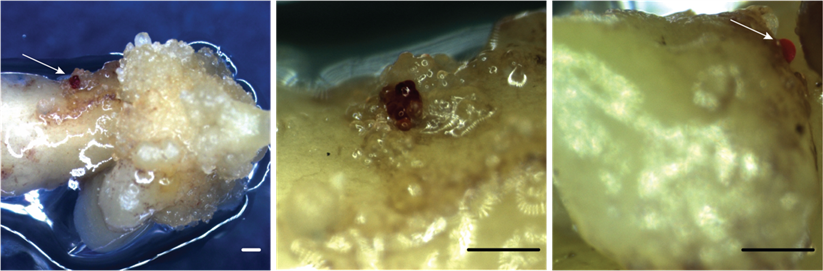


**Supplementary Figure S3.** *A. tumefaciens*-mediated genetic transformation of *P. sieboldii*. Somatic embryos expressing the *RUBY* reporter were formed after transformation with *A. tumefaciens* GV3101 harboring 35S:RUBY and cultured on callus-inducing medium. Arrows indicate the formation of transgenic somatic embryos. Scale bars = 1 mm.


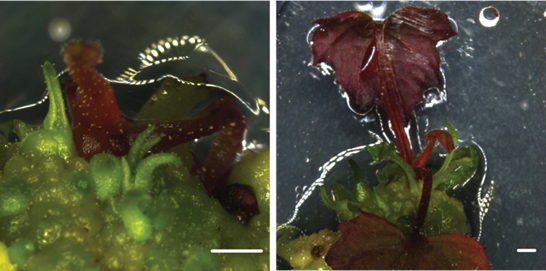


**Supplementary Figure S4.** Direct transgenic shoot formation after inoculation with *A. rhizogenes*. Transgenic shoots expressing the RUBY reporter formed directly from the embryogenic callus. Scale bars = 5 mm.


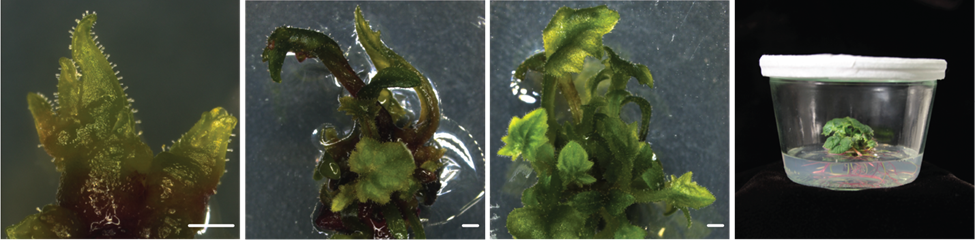


**Supplementary Figure S5.** Regeneration of *P. sieboldii* transgenic line TG6 from transformed hairy roots. Regenerated plants exhibit strong *RUBY* expression in roots, while leaves remain green. Scale bars = 1 mm.


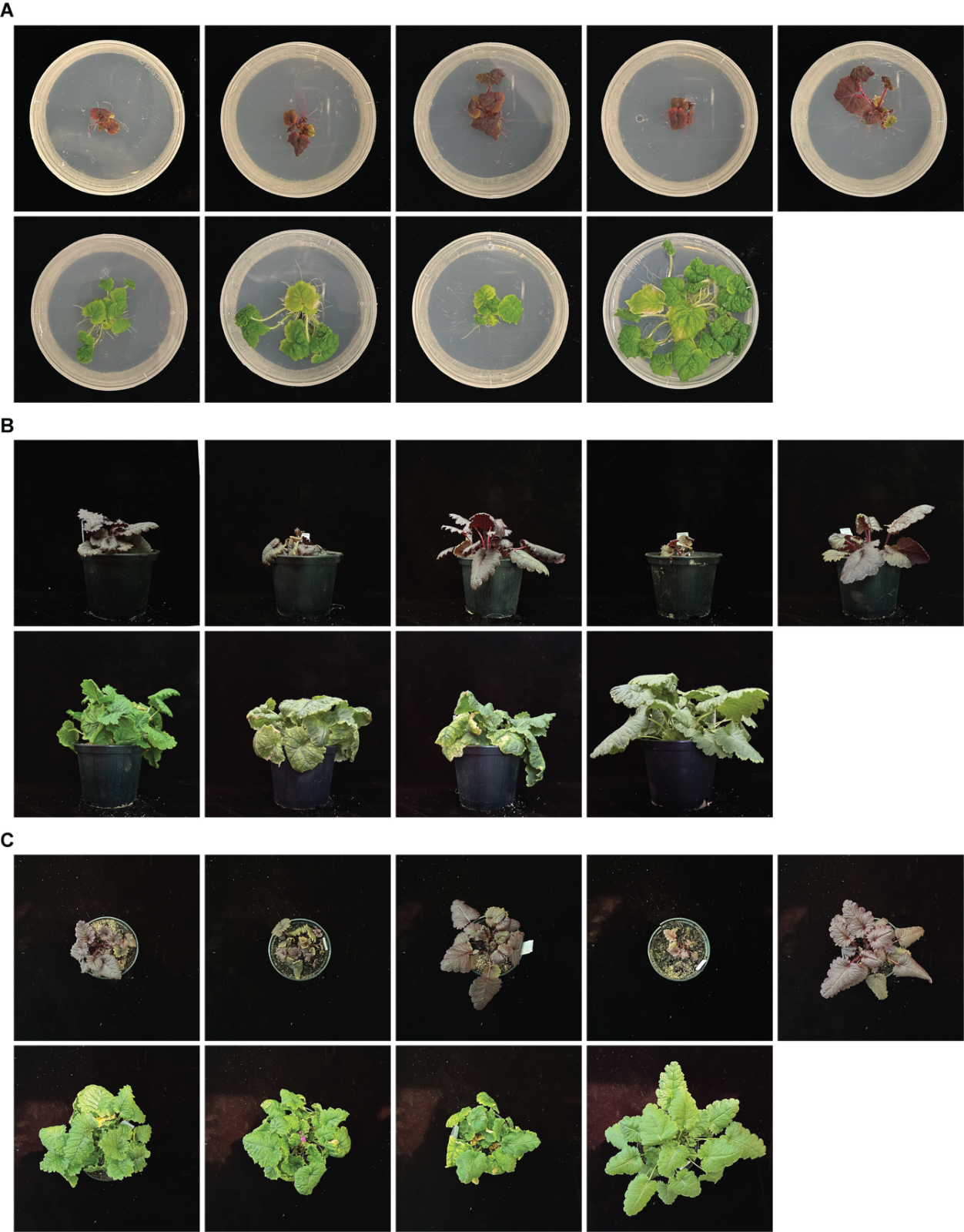

**Supplementary Figure S6.** Plant morphology of the T1 generation. (**A**) Plants growing *in vitro* 2.5 months after germination. (**B**, **C**) Plants growing in the greenhouse 5 months after transfer.


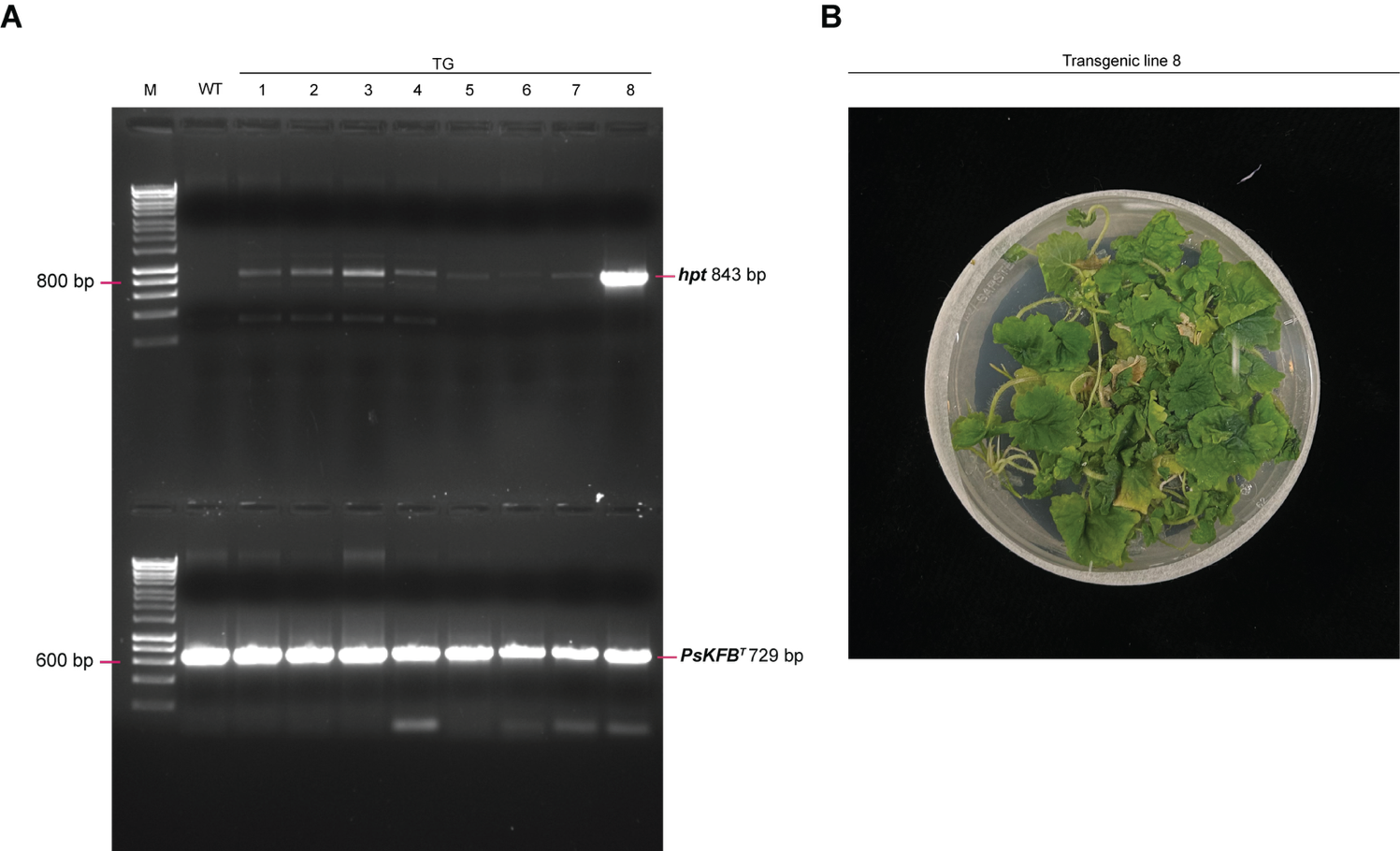

**Supplementary Figure S7.** PCR detection of transgenes in transformed *P. sieboldii*. M: marker, WT: wild-type *P. sieboldii* (S-morph), TG1–TG8: independent transgenic lines. (**A**) PCR was performed to detect the *hpt* gene from the pKG3 plasmid, and the *PsKFB^T^* gene located at the S-locus of *P. sieboldii*. The genotypes of donor plants (from which explants were taken) were confirmed prior to transformation to ensure they were S-morph. **(B)** PCR-positive for *hpt* transgene transgenic line 8, was derived from a transgenic hairy root induced after inoculation with *A. rhizogenes* A4.

**Supplementary Table S1. Sequencing depth and T-DNA/genome coverage ratio**

| **Transformant** | **Genome-wide coverage** | **T-DNA coverage** | **T-DNA / Genome coverage ratio** | **T-DNA insertion number** |
| --- | --- | --- | --- | --- |
| **TG1** | **3.8** | **4.3** | **1.13** | **Possibly two heterozygous insertions or a tandem insertion at one locus** |
| **TG3** | **5.2** | **4.1** | **0.79** | **Likely single heterozygous insertion** |
| **TG5** | **4.4** | **3.0** | **0.68** | **Likely single heterozygous insertion** |

**References**

Yang, L., Machin, F., Wang, S., Saplaoura, E., & Kragler, F. (2023). Heritable transgene-free genome editing in plants by grafting of wild-type shoots to transgenic donor rootstocks. *Nature biotechnology*, *41*(7), 958–967. https://doi.org/10.1038/s41587-022-01585-8
